# Social jetlag is associated with an increased likelihood of having depressive symptoms among the Japanese working population: the Furukawa Nutrition and Health Study

**DOI:** 10.1093/sleep/zsz204

**Published:** 2019-09-26

**Authors:** Zobida Islam, Huanhuan Hu, Shamima Akter, Keisuke Kuwahara, Takeshi Kochi, Masafumi Eguchi, Kayo Kurotani, Akiko Nanri, Isamu Kabe, Tetsuya Mizoue

**Affiliations:** 1 Department of Epidemiology and Prevention, Center for Clinical Sciences, National Center for Global Health and Medicine, Tokyo; 2 Teikyo University Graduate School of Public Health, Tokyo; 3 Department of Health Administration, Furukawa Electric Corporation, Tokyo; 4 Department of Nutritional Epidemiology and Shokuiku, Department of Nutritional Education, National Institute of Health and Nutrition, National Institutes of Biomedical Innovation, Health and Nutrition, Tokyo; 5 Department of Food and Health Sciences, International College of Arts and Sciences, Fukuoka Women’s University, Fukuoka, Japan

**Keywords:** cross-sectional study, social jetlag, depressive symptoms, Japanese, non-shift worker

## Abstract

**Study Objectives:**

Social jetlag, a mismatch between biological and social timing, has been reported to be associated with depressive symptoms among general population. However, evidence on this association is lacking among non-shift workers, who are under pressure to adapt themselves to a work schedule. We investigated the cross-sectional association of social jetlag with depressive symptoms among Japanese non-shift workers.

**Methods:**

This study included 1,404 employees, aged 18–78 years, who completed a study questionnaire at a periodic health checkup. Social jetlag was calculated as the absolute value of the difference in the midpoint of sleep times between weekdays and weekends. Depressive symptoms were assessed using the Center for Epidemiologic Studies Depression Scale. Multivariable logistic regression was used to estimate the odds ratio (OR) with adjustments for potential confounders including diet and chronotype.

**Results:**

Of the study participants, 63.5%, 28.4%, and 8.1% had less than 1 hour, 1 to less than 2 hours, and at least 2 hours of social jetlag, respectively. Greater social jetlag was significantly associated with an increased likelihood of having depressive symptoms. The multivariable-adjusted OR (95% confidence interval) were 1.30 (0.95 to 1.78) and 2.14 (1.26–3.62) for 1 to less than 2 hour and at least 2 hours compared to less than 1 hour of social jetlag. The association between social jetlag and depressive symptoms appeared to be linear, according to restricted cubic spline regression.

**Conclusion:**

Results suggest that greater social jetlag is associated with an increased likelihood of having depressive symptoms among non-shift workers.

**Statement of Significance**

Evidence linking depressive symptoms to social jetlag is lacking. We examined the cross-sectional association between social jetlag and depressive symptoms among Japanese non-shift workers. This study suggests that greater social jetlag was significantly associated with an increased likelihood of having depressive symptoms.

## Introduction

Depression is a leading cause of disability and a major contributor to suicide death [1]. Worldwide more than 300 million people have depression [1]. Clinical studies have revealed that patients with depressive symptoms show daily mood variations [2, 3], abnormal patterns of core body temperature [4], and hormone secretions such as cortisol and melatonin [5, 6] regulated by the endogenous circadian clock [7–9]. Accumulated evidence indicates that desynchronization of circadian timing leads to the loss of the internal temporal oscillations of cells and organs, as well as to hormonal and behavioral dysfunction, which, in turn, contributes to the onset of depression [10]. Social jetlag, a form of circadian desynchronization arising from alternating work and free days and leading to sleep and wakefulness at inappropriate biological times [11], has recently gained much attention. A meta-analysis of 11 prospective studies [12] reported that shift workers, who are well known for experiencing chronic social jetlag [13, 14], are at higher risk for depression, although the results were highly heterogenous (*I*^2^ = 78%).

Social jetlag is also common among the general population or non-shift workers. This is due to the considerable influence of social or work times on biological sleep timing [15, 16]. Evidence linking social jetlag and depression, however, is limited among these groups. To date, three cross-sectional studies (two Brazilian [17, 18] and one European [19]) addressed the association between social jetlag and depressive symptoms among students aged 12–21 years [17], the general adult population [18], and among patients with diagnosed states of major depressive disorders (MDD) and/or healthy controls [19]. Their results were conflicting—one study observed a positive association between social jetlag and depression [18], whereas others failed to confirm the association [17, 19]. From a methodological viewpoint, all these studies did not adjust for potentially important confounders including alcohol consumption, physical activity, sleep quality, and dietary intake (review [20, 21]). More importantly, in spite of the high prevalence of social jetlag among non-shift workers (85%) [16], no studies have been performed with this group.

We cross-sectionally evaluated the association between social jetlag and depressive symptoms among Japanese non-shift workers, while adjusting for a wide range of dietary and lifestyle-related factors.

## Materials and Methods

### Study design

Data for this study were derived from the Furukawa Nutrition and Health Study, the details of which have been described previously [22]. In brief, a health survey was conducted during periodic health examinations among employees of a manufacturing company and its affiliated companies in Chiba Prefecture and Kanagawa Prefecture, Japan. The survey was conducted at baseline (in April 2012 and May 2013) and at a 3-year follow-up session (in April 2015 and May 2016). This study was based on the follow-up survey, which had detailed information on sleep habits. Prior to the health checkup, participants were asked to fill out two types of questionnaires—one specially designed for diet and another for overall health-related lifestyle. In the second survey, 2,067 of the 2,350 who received a checkup agreed to participate in the survey (response rate: 88%). On the day of the health checkup, research staff checked the questionnaires for completeness and where necessary, clarified responses with the participants. We obtained health checkup data, including results of anthropometric and biochemical measurements, and disease history. The protocol for the study was approved by the ethics committee of the National Center for Global Health and Medicine, Japan.

### Participants

Of the survey participants, 107 employees with a history of cancer (*n* = 27), cardiovascular disease (*n* = 30), nephritis (*n* = 7), hepatitis (*n* = 2), pancreatitis (*n* = 2), and psychiatric disorders including depression and anxiety disorder (*n* = 39) were excluded. Some of the people excluded had two or more of these conditions. We excluded participants with a history of chronic diseases and psychiatric disorders because these conditions might affect both social jetlag and depressive symptoms. We then sequentially excluded those who lacked information on bedtimes and wake-up times (*n* = 21), depression status and covariates used in the present analysis (*n* = 19). We further excluded night time workers (*n* = 171) and rotating shift workers (*n* = 344). This left 1,404 participants (1,191 men and 213 women), aged between 18 and 78 years, for the social jetlag and depression analysis.

### Social jetlag

Social jetlag status was determined using questions about typical bedtime, sleep latency, and wake times on weekdays and weekends. The midpoint of sleep was assessed by the sleep onset time (adding sleep latency time to bedtime) and wake time. It was then calculated for each weekday (MSW) and weekend days or free days (MSF). Following the formula established by Wittman *et al.* [11], social jetlag was estimated as the absolute value of the differences (in hours) in the midpoint of sleep times between weekdays and weekends (MSF − MSW). In this study, social jetlag ranged from 0 to 4.5 hours. We thus classified participants into three groups namely, less than 1 hour of social jetlag, 1 to less than 2 hours of social jetlag, and at least 2 hours of social jetlag. The reference category was less than 1 hour as done in previous studies [15, 19].

### Depressive symptoms

Depressive symptoms were assessed using a Japanese version [23] of the Center for Epidemiologic Studies Depression (CES-D) Scale [24]. This scale consisted of 20 items covering six typical symptoms of depression: depressed mood, feelings of guilt or worthlessness, feelings of helplessness or hopelessness, psychomotor retardation, loss of appetite, and sleep disturbance experienced during the preceding week. Each item was scored on a scale of 0–3 according to the frequency of the symptoms and summed up to the total CES-D score, ranging from 0 to 60. The criterion validity of the CES-D Scale has been well established in both Western [24] and Japanese [23] individuals. Prevalent cases of depressive symptoms had a CES-D score of at least 16.

### Other variables

Sleep duration, sleep quality, and chronotype were taken into account because these variables have been linked with social jetlag and depressive symptoms [18, 25]. Chronotype was estimated using mid-sleep time on free days corrected for sleep debt on workdays (MSFsc). We applied an algorithm, which was proposed by Roenneberg *et al.* [26], to assess MSFsc: MSFsc = MSF − 0.5 × [SDF − (5 × SDW + 2 × SDF)/7]. Here, SDW is sleep duration on workdays and SDF is sleep duration on free days. MSF is the midpoint of sleep on free days. MSFsc provides a quantitative measure of chronotype as a continuous variable [26]. We assessed sleep quality by asking participants to rate overall sleep quality using the following response options: very good, good, not so good, or bad. The question and response options were similar to one of the questions in the Japanese version of the Pittsburgh Sleep Quality Index [27]. We calculated the weighted average sleep duration (weight of “five” assigned to weekdays and “two” assigned to weekends) using typical bedtime, sleep latency, and wake times on weekdays and weekends. As social jetlag differs across age and sex [28], these variables were included as covariates in our analysis. As social zeitgebers, such as meals, exercise, or social demands (i.e. work) serves to entrain human’s biological rhythm (i.e. sleep) [29], potential influence of such external factors on biological clock should be taken into account. Therefore, we included work site, job, job grade, flexible time work, overtime work, marital status, smoking status, alcohol consumption, work-related physical activities, leisure-time physical activities, and body mass index (BMI) as covariates. Body height and weight were measured to the nearest 0.1 cm and 0.1 kg, respectively with participants wearing light clothes and no shoes. BMI was calculated as the body weight in kilograms divided by the square of the body height in meters. Job, job grade, flexible time work, marital status, overtime, smoking status, alcohol consumption, work-related physical activities, and leisure-time physical activities were assessed via a lifestyle questionnaire. Work-related and leisure-time physical activities were each expressed as the sum of their metabolic equivalent (MET) value multiplied by the duration of that activity. As dietary intake of potential nutrients is related to both social jetlag [30] and depressive symptoms [21, 31], these variables were also considered in our analysis. Diet was assessed via a validated self-administered questionnaire. Energy and selected nutrients were estimated using an ad hoc computer algorithm with reference to the Standard Tables of Food Composition in Japan [32].

### Statistical analysis

Proportions and means were presented to show the background characteristics of the study population according to the categories of social jetlag. The trend association between potentially confounding variables and social jetlag was assessed using the Mantel–Haenszel chi-square test for categorical variables and linear regression analysis for continuous variables, respectively. Multivariable logistic regression analyses were performed to estimate odds ratios (ORs) and 95% confidence intervals (CIs) for investigating the association of depression with social jetlag. In the first model, we adjusted for age (year, continuous), sex, and site. In the second model, we made additional adjustments for job (white-collar or blue-collar worker), marital status (yes or no), overtime (<10, 10–29, or ≥30 h/mo), smoking status (never-smoked, former smoker, current smoker consuming <20 cigarettes/d, or current smoker consuming ≥20 cigarettes/d), alcohol consumption (nondrinker including those consuming alcohol less than once per week, drinkers consuming <23 g, drinkers consuming ≥23 g to <46 g, or drinkers consuming ≥46 g of ethanol/d), BMI (kg/m^2^), flexible time work (yes or no), average sleep duration (hours per day, continuous), chronotype (hours, continuous), and sleep quality (good, very good, not so good, or bad). In the third model, we further adjusted for physical activity at work (<3, 3 to <7, 7 to <20, or ≥20 METs-h/d), leisure-time physical activities (0, 0 to <3, 3 to <10, or ≥10 METs-h/wk), energy intake (kcal/d, continuous), intake of magnesium (mg/1,000 kcal, continuous), calcium (mg/1,000 kcal, continuous), iron (mg/1,000 kcal, continuous), zinc (mg/1,000 kcal, continuous), folate (µg/1,000 kcal, continuous), vitamin C (mg/1,000 kcal, continuous), vitamin B6 (mg/1,000 kcal, continuous), vitamin B12 (µg/1,000 kcal, continuous), and ω-3 polyunsaturated fatty acids (% energy, continuous). Trend association was assessed by assigning ordinal numbers to the categories of social jetlag, treating them as a continuous variable. To investigate the consistency of our findings, we assessed the association by including social jetlag as a continuous term. We additionally performed multivariable linear regression analysis to investigate whether social jetlag is associated with continuous depression severity scores (CES-D scores). To examine whether social jetlag is associated with depressive symptoms independently of chronotype difference, we performed sensitivity analysis after excluding participants with extreme early and late type of chronotype. The criteria for extreme early and late types of chronotype were defined 2.5% at each end of the MSFsc distribution as done in previous studies [33, 34]. We also performed the restricted cubic spline regression to evaluate the shape of the relation between social jetlag and the odds of depressive symptoms by assigning 37.5 minutes to the reference value of social jetlag and 0, 37.5, and 105 minutes (10th, 50th, and 90th percentiles), respectively to the three knots. Two-sided *p* values (<0.05) were regarded as statistically significant. All analyses were performed using statistical software Stata version 14 (StataCorp, College Station, Texas).

## Results

Table 1 shows the characteristics of study participants according to the categories of social jetlag. The proportion of those with less than 1 hour, 1 to less than 2 hours and at least 2 hours of social jetlag were 63.5%, 28.4%, and 8.1%, respectively. Subjects with greater social jetlag were younger and more likely to be female, in a low-ranking job position, blue-collar workers, a current smoker, and tended to have late type of chronotype, a higher CES-D scores, and a poor quality of sleep. They were less likely to be married, a current alcohol drinker, and physically active at work and during leisure time. Regarding diet, the participants with greater social jetlag tended to consume lower amounts of vitamin C, calcium, magnesium, iron, and folate.

**Table:** Table 1. Participants characteristic according to the categories of social jetlag

	Social jetlag			
Participants characteristics	<1 h	1 to <2 h	≥2 h	*P*-value* for trend
Number of subjects	892	399	113	
Age (mean ± *SD*, y)	46.2 ± 9.4	42.9 ± 9.6	39.7 ± 10.8	<0.001
Sex (women, %)	13.3	17.8	20.4	0.009
Work (survey in April 2015, %)	52.9	54.1	54.9	0.61
Flexible time work (yes, %)	18.2	22.6	19.5	0.20
Marital status (married, %)	75.0	60.9	39.8	<0.001
Employment type (regular, %)	91.1	94.0	90.3	0.47
Blue-collar workers (%)	29.2	35.9	40.7	<0.001
Job grade (low, %)	83.6	88.5	92.9	<0.001
Overtime (≥30 h/mo, %)	22.3	22.1	19.5	0.58
Current smoker (%)	23.5	29.3	34.5	0.002
Current alcohol drinker (≥1 d/wk, %)	56.3	44.6	48.7	<0.001
Work-related physical activity (≥20 METs-h/d, %)	14.2	21.1	20.3	0.003
Leisure-time physical activity (≥10 METs-h/wk, %)	29.4	24.1	18.6	0.004
BMI (kg/m^2^)	23.5 ± 3.4	23.4 ± 3.7	23.9 ± 4.0	0.66
Dietary intake (per d)				
Total energy (mean ± *SD*, kcal)	1819 ± 508	1768 ± 545	1770 ± 613	0.11
Magnesium (mg/1,000 kcal)	128 ± 25	124 ± 26	122 ± 27	0.002
Calcium (mg/1,000 kcal)	248 ± 91	230 ± 85	228 ± 103	<0.001
Iron (mg/1,000 kcal)	3.9 ± 1.0	3.8 ± 1.0	3.7 ± 1.0	0.02
Zinc (mg/1,000 kcal)	4.2 ± 0.6	4.2 ± 0.67	4.2 ± 0.7	0.09
Folate (µg/1,000 kcal)	163 ± 57	157 ± 56	151 ± 50	0.008
Vitamin C (mg/1,000 kcal)	49 ± 23	48 ± 24	44 ± 22	0.04
Vitamin B_6_ (mg/1,000 kcal)	0.63 ± 0.19	0.62 ± 0.22	0.60 ± 0.23	0.21
Vitamin B_12_ (µg/1,000 kcal)	4.7 ± 2.3	4.5 ± 2.3	4.5 ± 2.6	0.25
ω-3 PUFA (% energy)	1.2 ± 0.4	1.2 ± 0.4	1.2 ± 0.4	0.07
Sleep duration (mean ± *SD*, h/d)	6.5 ± 0.9	6.5 ± 0.8	6.7 ± 1.1	0.22
Average sleep quality (bad, %)	32.3	39.1	43.4	0.002
Chronotype (mean ± *SD*, h:min)	2:56 ± 0.58	3:52 ± 0.57	4:54 ± 0.95	<0.001
CES-D score [median (IQR)]	11 (7–16)	12 (8–17)	14 (10–20)	<0.001

IQR, Interquartile range; PUFA, polyunsaturated fatty acids.

*Based on Mantel–Haenszel chi-square test for categorical variables and linear regression analysis for continuous variables.

As shown in Table 2, 422 (30.1 %) participants were identified as having depressive symptoms. Greater social jetlag was significantly associated with the increased likelihood of having depressive symptoms in model 1 (*p-*trend <0.001). In model 2 with adjustments for lifestyle-related covariates other than physical activity and dietary factors, the OR (95% CI) of having depressive symptoms from the lowest (<1 h) through to the highest (≥2 h) category of social jetlag was 1.00 (reference), 1.33 (0.98 to 1.81), and 2.32 (1.39 to 3.87), respectively (*p-*trend = 0.002). The association was only slightly attenuated and remained statistically significant after adjustment for physical activity at work and leisure time, and dietary-related covariates (model 3). The OR (95% CI) of having depressive symptoms from the lowest (<1 h) through to the highest (≥2 h) category of social jetlag was 1.00 (reference), 1.30 (0.95 to 1.78), and 2.14 (1.26 to 3.62), respectively (*p-*trend = 0.01). The association between social jetlag and depressive symptoms virtually unchanged after excluding participants with morning and late type of chronotype (*n* = 73). The multivariable adjusted OR (95% CI) of having depressive symptoms was 1.20 (0.90 to 1.62) and 1.83 (1.09 to 3.07) for the participants with 1 to less than 2 and at least 2 hours of social jetlag compared to the participants with less than 1 hour of social jetlag (Supplementary Table S1). Similar association was obtained when social jetlag was considered as a continuous variable; the multivariable-adjusted OR (95% CI) for per hour of social jetlag was 1.35 (1.09 to 1.67) (Supplementary Table S2). Considering depression score (CES-D score) as a continuous variable, participant with at least 2 hours of social jetlag showed increased depression severity score compared to participants with less than 1 hour of social jetlag in model 1 (*p* < 0.001). After additional adjustment for all the covariates (model 2 and model 3), the association was attenuated and became statistically nonsignificant (Supplementary Table S3).

**Table:** Table 2. Multivariable-adjusted OR and 95% CI for the association of social jetlag with depressive symptoms

	Social jetlag			
	<1 h	1 to <2 h	≥ 2 h	*P*-value for trend*
CES-D (≥16)				
Cases/number of subjects	238/892	132/399	52/113	
Model 1^†^	1.00 (reference)	1.29 (1.00 to 1.68)	**2.13 (1.41 to 3.20)**	**<0.001**
Model 2^‡^	1.00 (reference)	1.33 (0.98 to 1.81)	**2.32 (1.39 to 3.87)**	**0.002**
Model 3^§^	1.00 (reference)	1.30 (0.95 to 1.78)	**2.14 (1.26 to 3.62)**	**0.01**

Values in bold show statistical significance.

*Based on multivariable logistic regression analysis with assignment of ordinal numbers to each category of social jetlag.

^†^Model 1 adjusted for age (year, continuous), sex, and site.

^‡^Model 2 additionally adjusted for job (white-collar or blue-collar worker), job grade (low or middle and high), married (yes or no), overtime work (<10, 10–29, or ≥30 h/mo), smoking status (never-smoked, former smoker, current smoker smoking <20 cigarettes/d, or current smoker smoking ≥20 cigarettes/d), alcohol consumption (nondrinker including infrequent drinker consuming alcohol less than once per week, drinker consuming <23 g of ethanol/d, drinker consuming ≥23 to <46 g of ethanol/d, or drinker consuming ≥46 g of ethanol/d), BMI (kg/m^2^, continuous), average sleep duration on weekdays and on the weekend (hours/day, continuous), sleep quality (good, very good, not so good, or bad), flexible work (yes or no), and chronotype (hours, continuous).

^§^Model 3 additionally adjusted for physical activity at work (<3, 3 to <7, 7 to <20, or ≥20 METs-h/d), leisure-time physical activities (0, 0 to <3, 3 to <10, or ≥10 METs-h/wk), energy intake (kcal/day, continuous), intake of magnesium (mg/1,000 kcal, continuous), calcium (mg/1,000 kcal, continuous), iron (mg/1,000 kcal, continuous), zinc (mg/1,000 kcal, continuous), folate (µg/1,000 kcal, continuous), vitamin C (mg/1,000 kcal, continuous), vitamin B_6_ (mg/1,000 kcal, continuous), vitamin B_12_ (µg/1,000 kcal, continuous), and ω-3 PUFA (% energy, continuous).

**Figure 1.**
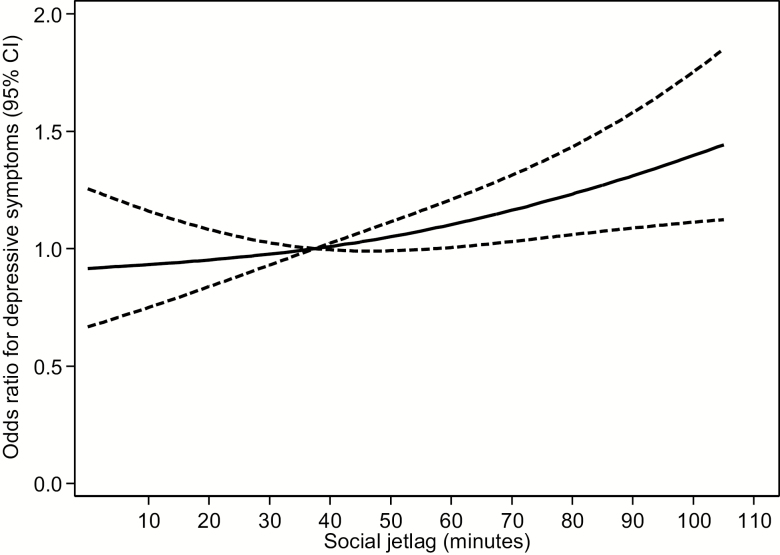
Restricted cubic spline regression for the association between the duration of social jetlag and depressive symptoms. The solid line represents the OR and the dashed line represents the 95% CI. Knots were placed at the 10th, 50th, and 90th percentiles (0, 37.5, and 105 min, respectively) of social jetlag. The reference value was 37.5 min. The model was adjusted for age (year, continuous), sex, site, job (white-collar or blue-collar worker), job grade (low or middle and high), married (yes or no), overtime (<10, 10–29, or ≥30 h/mo), smoking status (never-smoked, former smoker, current smoker smoking <20 cigarettes/d, or current smoker smoking ≥20 cigarettes/d), alcohol consumption (nondrinker including infrequent drinker consuming alcohol less than once per week, drinker consuming <23 g of ethanol/d, drinker consuming ≥23 to <46 g of ethanol/d, or drinker consuming ≥46 g of ethanol/d), BMI (kg/m^2^, continuous), average sleep duration on weekdays and on the weekend (hours/day, continuous), sleep quality (good, very good, not so good, or bad), flexible work (yes or no), chronotype (hours, continuous), physical activity at work (<3, 3 to <7, 7 to <20, or ≥20 METs-h/d), leisure-time physical activities (0, 0 to <3, 3 to <10, or ≥10 METs-h/wk), energy intake (kcal/day, continuous), intake of magnesium (mg/1,000 kcal, continuous), calcium (mg/1,000 kcal, continuous), iron (mg/1,000 kcal, continuous), zinc (mg/1,000 kcal, continuous), folate (µg/1,000 kcal, continuous), vitamin C (mg/1,000 kcal, continuous), vitamin B_6_ (mg/1,000 kcal, continuous), vitamin B_12_ (µg/1,000 kcal, continuous), ω-3 PUFA (% energy, continuous).

According to the results of the cubic spline regression analysis (Figure 1), the association between the duration of social jetlag and depressive symptoms (model 3) appeared to be linear. The odds of depressive symptoms steadily increased with the increasing severity of social jetlag.

## Discussion

In this cross-sectional study, we found that greater social jetlag was significantly associated with a higher prevalence of depressive symptoms, even after adjusting for a range of potential confounders. We also observed a linear relationship between social jetlag and depressive symptoms using a restricted cubic spline regression analysis. To the best of our knowledge, this is the first study to investigate the association between social jetlag and depressive symptoms in non-shift workers.

Of three previous cross-sectional studies on this issue [17–19], the present findings are consistent with the results of a study conducted by Levandovski *et al.* [18]. This study showed a significant positive association between social jetlag and depressive symptoms (assessed using the Beck Depression Inventory) among 4,051 Southern Brazilian adults aged 18–65 years [18]. In contrast, no association was observed in the studies on Southern Brazilian students (aged 12–21 years) [17], and on clinically depressed patients and healthy controls in the Netherlands [19]. The reason for the null association in these studies is not clear. In young students, depressive symptoms may be induced by factors other than circadian misalignment, including puberty-, hormones-, and sexual- or emotional abuse-related factors [35]. The use of antidepressant medication among patients with depression may mask the effect of social jetlag [19]. All those previous studies [17–19] did not adjust for potentially important confounders such as physical activity and diet. With adjustment for a wide range of potentially important confounders, this study adds evidence to support an independent role of social jetlag in depressive symptoms.

In the analysis using restricted cubic regression (a more suitable technique in assessing the shape of the association), we found a linear relationship between social jetlag and depressive symptoms. To date, no study has assessed the shape of the association between social jetlag and depressive symptoms. A cross-sectional study among women from the United States with MDD (aged 19–60 years) reported a linear relationship between circadian misalignment (misalignment between the timing of the biological clock and the timing of sleep) and the severity of depression symptoms [36]. Findings from this study indicate that the more severe the social jetlag, the higher the probability of having depressive symptoms.

Precise mechanism linking social jetlag and depression is unclear. As social jetlag is a measure of the discrepancy between endogenous biological timing and external timing, it is possible that circadian disruption underlies this association. A number of studies show that circadian disruption plays a crucial role in the pathogenesis of depression [10, 26, 37]. Disturbance in the circadian sleep–wake cycle exacerbates the depressive state owing to altered patterns in the secretion of monoamine neurotransmitters, such as serotonin, noradrenalin, and dopamine, which regulate mood and are involved in the pathophysiology of depression (review [10]). Human experimental studies have shown that disruption in biological sleep timing affects the circadian endocrine function and results in a decreased mean 24-hour melatonin level [38] and an altered pattern of 24-hour norepinephrine and cortisol rhythm [39]. This, in turn, contributes to the development of depressive symptoms [40–42]. Furthermore, a human experimental study observed that misalignment in biological sleep–wake timing elevates the plasma level of high sensitivity C-reactive protein [43], which has been associated with the increased risk of psychological distress and depression in the general population [44].

The major strengths of this study include the high participation rate and adjustment for a range of potentially confounding variables. Another strength is the assessment of sleep onset time. Taking the sleep latency period into account makes the quantification of social jetlag more precise. We also acknowledged some limitations in this study. First, an association observed in a cross-sectional study does not necessarily indicate causality. Depression as well as depressive mood could disrupt the normal or regular sleep patterns [45]. Therefore, workers with depressive symptoms may be more likely to have irregular sleep timing and may tend to sleep longer on weekends to compensate for their sleep loss over the week leading to social jetlag. To reduce the possibility of reverse causality, we excluded subjects with a history of mental disorders that might affect social jetlag and depressive symptoms. Second, social jetlag was estimated via a self-reported questionnaire, which is subject to reporting bias and may not provide an accurate sleep pattern. Third, although we adjusted for numerous potential confounders, we could not rule out the possibility that the observed associations are due to unmeasured and residual confounding. For example, we assessed overall sleep quality using a single question, which may not have accurately measured sleep quality. Therefore, this could be a source of residual confounding. Finally, the study subjects were non-shift workers at a manufacturing company. The present findings thus may not be generalizable to the unemployed, shift workers, or non-shift workers with different backgrounds.

## Conclusions

Social jetlag was associated with an increased likelihood of having depressive symptoms among Japanese non-shift workers. This study extended evidence regarding social jetlag and depressive symptoms to the Asian working population. Workers more likely to adopt working schedules and less likely to maintain a regular sleep timing, will experience chronic social jetlag throughout their working career. Our findings from this occupational setting stress the beneficial effects of maintaining regularity in the sleep–wake cycle for depressive symptoms. Prospective studies are warranted to confirm the present cross-sectional observation.

## Supplementary Material

zsz204_suppl_Supplementary_Tables
