# Supplementary material for: Social jetlag is associated with an increased likelihood of having depressive symptoms among the Japanese working population: the Furukawa Nutrition and Health Study
Source: Sleep. 2019 Sep 26;43(1):zsz204. doi: 10.1093/sleep/zsz204 (PMC6985924; doi:10.1093/sleep/zsz204)
Supplement: zsz204_suppl_Supplementary_Tables [file zsz204_suppl_supplementary_tables.docx]

**Table S1** Multivariable-adjusted odds ratio and 95% confidence interval for the association of social jetlag with depressive symptoms after excluding the participants with early and late type of chronotype

**Table S2** Multivariable-adjusted odds ratio and 95% confidence interval for the association of social jetlag (continuous) with depressive symptoms.

**Table S3** Multivariable linear regression analysis of the relationship between social jetlag and continuous CES-D score (log-tranfromed).

**Table S1** Multivariable-adjusted odds ratio and 95% confidence interval for the association of social jetlag with depressive symptoms after excluding the participants with early and late type of chronotype

| CES-D scores (≥16) | Social jetlag | | | *P* for trend^a^ |
| --- | --- | --- | --- | --- |
|  | <1 hour | 1 to <2 hours | ≥2 hours |  |
| Cases/number of subjects | 225/857 | 127/388 | 39/86 |  |
| Model 1^b^ | 1.00 (reference) | 1.31 (1.00-1.71) | 2.14 (1.35-3.39) | 0.001 |
| Model 2^c^ | 1.00 (reference) | 1.25 (0.93-1.67) | 1.97 (1.18-3.26) | 0.007 |
| Model 3^d^ | 1.00 (reference) | 1.20 (0.90-1.62) | 1.83 (1.09-3.07) | 0.02 |

Abbreviation: CES-D, Center for Epidemiologic Studies Depression Scale. ^a^Based on multivariable logistic regression analysis with assignment of ordinal numbers to each category of social jetlag. ^b^Model 1 adjusted for age (year, continuous), sex, and site. ^c^Model 2 additionally adjusted for job (white-collar or blue-collar worker), job grade (low or middle and high), married (yes or no), overtime work (<10 hours/month, 10-29 hours/month, or $\geq$30 hours/month), smoking status (never-smoked, former smoker, current smoker smoking <20 cigarettes/day, or current smoker smoking ≥20 cigarettes/day), alcohol consumption (nondrinker including infrequent drinker consuming alcohol less than once per week, drinker consuming <23 g of ethanol/day, drinker consuming$\geq$23-<46 g of ethanol/day, or drinker consuming $\geq$46 g of ethanol/day), BMI (kg/m^2^, continuous), average sleep duration on weekdays and on the weekend (hours/day, continuous), sleep quality (good, very good, not so good, or bad), and flexible work (yes or no). ^d^Model 3 additionally adjusted for physical activity at work (<3 METs-hours/day, 3-<7 METs-hours/day, 7-<20 METs-hours/day, or ≥20 METs-hours/day), leisure-time physical activities (0 METs-hours/week, 0-<3 METs-hours/week, 3-<10 METs-hours/week, or ≥10 METs-hours/week), energy intake (Kcal/day, continuous), intake of magnesium (mg/1000 kcal, continuous), calcium (mg/1000 kcal, continuous), iron (mg/1000 kcal, continuous), zinc (mg/1000 kcal, continuous), folate (µg/1000 kcal, continuous), vitamin C (mg/1000 kcal, continuous), vitamin B6 (mg/1000 kcal, continuous), vitamin B12 (µg/1000 kcal, continuous), and ω-3 PUFA (% energy, continuous).

**Table S2** Multivariable-adjusted odds ratio and 95% confidence interval for the association of social jetlag (continuous) with depressive symptoms

| CES-D (≥ 16) | social jetlag per hour | *P* for trend |
| --- | --- | --- |
| Model 1^a^ | 1.37 (1.18-1.60) | **<0.001** |
| Model 2^b^ | 1.40 (1.14-1.73) | **0.001** |
| Model 3^c^ | 1.35 (1.09-1.67) | **0.005** |

Abbreviation: CES-D, Center for Epidemiologic Studies Depression Scale.

^a^Model 1 adjusted for age (year, continuous), sex, and site. ^b^Model 2 additionally adjusted for job (white-collar or blue-collar worker), job grade (low or middle and high), married (yes or no), overtime work (<10 hours/month, 10-29 hours/month, or $\geq$30 hours/month), smoking status (never-smoked, former smoker, current smoker smoking <20 cigarettes/day, or current smoker smoking ≥20 cigarettes/day), alcohol consumption (nondrinker including infrequent drinker consuming alcohol less than once per week, drinker consuming <23 g of ethanol/day, drinker consuming$\geq$23-<46 g of ethanol/day, or drinker consuming $\geq$46 g of ethanol/day), BMI (kg/m^2^, continuous), average sleep duration on weekdays and on the weekend (hours/day, continuous), sleep quality (good, very good, not so good, or bad), flexible work (yes or no), and chronotype (hours, continuous). ^c^Model 3 additionally adjusted for physical activity at work (<3 METs-hours/day, 3-<7 METs-hours/day, 7-<20 METs-hours/day, or ≥20 METs-hours/day), leisure- time physical activities (0 METs-hours/week, 0-<3 METs-hours/week, 3-<10 METs-hours/week, or ≥10 METs-hours/week), energy intake (Kcal/day, continuous), intake of magnesium (mg/1000 kcal, continuous), calcium (mg/1000 kcal, continuous), iron (mg/1000 kcal, continuous), zinc (mg/1000 kcal, continuous), folate (µg/1000 kcal, continuous), vitamin C (mg/1000 kcal, continuous), vitamin B6 (mg/1000 kcal, continuous), vitamin B12 (µg/1000 kcal, continuous), and ω-3 PUFA (% energy, continuous).

**Table S3** Multivariable linear regression analysis of the relationship between social jetlag and continuous CES-D score (log-tranfromed)

|  | *β* coefficient (95% CI) | | |
| --- | --- | --- | --- |
|  | Model 1^a^ | Model 2^b^ | Model 3 ^c^ |
| **Social jetlag** |  |  |  |
| <1 hour | 0.00 (reference) | 0.00 (reference) | 0.00 (reference) |
| 1 to <2 hours | 0.16 (0.55 - 0.27) | 0.13 (0.02 – 0.24) | 0.11 (- 0.001 – 0.22) |
| ≥2 hours | 0.26 (0.08 – 0.44) | 0.19 (-0.01 – 0.38) | 0.13 (- 0.06 – 0.33) |
| *P* for trend | **<0.001** | **0.01** | 0.05 |

Abbreviation: CES-D, Center for Epidemiologic Studies Depression Scale. ^a^Model 1 adjusted for age (year, continuous), sex, and site. ^b^Model 2 additionally adjusted for job (white-collar or blue-collar worker), job grade (low or middle and high), married (yes or no), overtime work (<10 hours/month, 10-29 hours/month, or $\geq$30 hours/month), smoking status (never-smoked, former smoker, current smoker smoking <20 cigarettes/day, or current smoker smoking ≥20 cigarettes/day), alcohol consumption (nondrinker including infrequent drinker consuming alcohol less than once per week, drinker consuming <23 g of ethanol/day, drinker consuming$\geq$23-<46 g of ethanol/day, or drinker consuming $\geq$46 g of ethanol/day), BMI (kg/m^2^, continuous), average sleep duration on weekdays and on the weekend (hours/day, continuous), sleep quality (good, very good, not so good, or bad), flexible work (yes or no), and chronotype (hours, continuous). ^c^Model 3 additionally adjusted for physical activity at work (<3 METs-hours/day, 3-<7 METs-hours/day, 7-<20 METs-hours/day, or ≥20 METs-hours/day), leisure- time physical activities (0 METs-hours/week, 0-<3 METs-hours/week, 3-<10 METs-hours/week, or ≥10 METs-hours/week), energy intake (Kcal/day, continuous), intake of magnesium (mg/1000 kcal, continuous), calcium (mg/1000 kcal, continuous), iron (mg/1000 kcal, continuous), zinc (mg/1000 kcal, continuous), folate (µg/1000 kcal, continuous), vitamin C (mg/1000 kcal, continuous), vitamin B6 (mg/1000 kcal, continuous), vitamin B12 (µg/1000 kcal, continuous), and ω-3 PUFA (% energy, continuous).
